# Unveiling the Role Displayed by *Penicillium digitatum PdMut3* Transcription Factor in Pathogen–Fruit Interaction

**DOI:** 10.3390/jof7100828

**Published:** 2021-10-03

**Authors:** Marta de Ramón-Carbonell, Paloma Sánchez-Torres

**Affiliations:** Instituto Valenciano de Investigaciones Agrarias (IVIA), Centro de Protección Vegetal y Biotecnología, 46113 Moncada, Spain; mardera@yahoo.es

**Keywords:** citrus, fungicide resistance, *Penicillium digitatum*, postharvest, transcription factor, virulence, peroxisomes

## Abstract

Zn_2_Cys_6_ transcription factors are unique to fungi and are involved in different regulatory functions. In this study, we have identified the *Penicillium digitatum* *PdMut3* gene, which encodes a putative Zn (II) 2Cys6 DNA-binding protein. Elimination of *PdMut3* in Pd1 strain caused increased virulence during citrus infection. The transcription of the *PdMut3* gene showed a higher expression rate during fungal growth and less transcription during fruit infection. Furthermore, the deletion of the gene in the wild-type isolate of *P. digitatum* did not produce any modification of the sensitivity to different fungicides, indicating that the gene is not associated with resistance to fungicides. In contrast, *PdMut3* null mutants showed a reduction in growth in minimal media, which was associated with severe alterations in conidiophore development and morphological alterations of the hyphae. Mutants showed greater sensitivity to compounds that interfere with the cell wall and an invasive growth block. Thus, *PdMut3* might have an indirect role in fungi virulence through metabolism and peroxisomes development.

## 1. Introduction

During the plant–pathogen interaction, numerous reactions are triggered, allowing the progression of the infection by the pathogen. In citrus fruits, *Penicillium digitatum*, the main pathogen during the postharvest period, is responsible for green mold that can lead to up to 90% losses [1]. To afford new control actions in postharvest pathogens, it is necessary to understand the regulatory mechanisms that govern processes involved in fungal–plant interaction. These processes comprise germination, mycelial progression, pathogenesis/virulence, host-specificity, or fungicide resistance in order to perform an effective infection.

Usually, pathogens displayed various mechanisms to rise their virulence [2]. During pathogen–host interaction, the start point of primary infection is wounds on the surface of the fruit, where different compounds promote the germination of spores, followed by diffusion and colonization of the fruit tissue [3]. There are more and more studies involved in processes related to virulence in postharvest pathogenic fungi [4]. Progresses in fungal pathogenicity and fruit response have recently been reported in *P. digitatum* [5]. Many of these studies reveal the genes and molecular mechanisms underlying the infection and the increase in pathogen virulence [5,6,7,8,9,10,11,12].

In a broad sense, virulence in plant pathogenic fungi is regulated by a system of cellular pathways that react to signals faced during host infection. Despite the variety of fungi and forms of infection, the signaling components that control pathogenic progress are basically preserved [13].

Transcription factors (TFs) are proteins that control gene expression by binding to a specific DNA sequence in the promoter region. Zinc finger TFs are one of the largest groups of transcriptional regulators, many of which have been characterized [14]. Zinc cluster proteins constitute one of the broadest families of transcription regulators in eukaryotes, can act as multifunctional regulators in many biological processes, and contribute to numerous functions in both transcription and translation procedures [15]. Previous studies showed that Zn_2_C_6_ transcription factors participate in primary metabolism, including sugar, amino acid, vitamin, and uracil metabolism [16], and in secondary metabolism, such as ergosterol biosynthesis and melanin biosynthesis [17]. Additionally, Zn_2_C_6_ transcription factors can contribute to fungal development and in the response to stresses, such as heat shock, oxidative stress and high osmotic stress [18,19], and pleiotropic drug resistance [20].

Zinc finger family TFs are classified into three main classes based on the number and order of cysteine residues: Cys2His2 proteins, Cys4 zinc finger proteins, and Zn2Cys6 proteins [21]. The third type is a large transcription factor family unique to fungi. The members of this family possess a Cys6 signature sequence and coordinate two zinc atoms per monomer [22], giving rise to the so-called zinc-binding nucleus Zn (II) 2Cys6 (abbreviated Zn_2_C_6_). This third class includes many of the most relevant transcription factors and includes the Gal4p transcription factor, which is possibly the best known and most studied zinc cluster protein in *Saccharomyces cerevisiae* [21].

This group includes the Zn_2_Cys_6_ transcription factors that are unique in the fungal regulatory network and some of them, such as GPF1 and CNF2 TFs, that have been functionally analyzed by genetic transformation in *Magnaporthe oryzae* are required for virulence [14]. Another example reported is Fow2, a Zn(II)2Cys6-type transcription regulator, which controls the expression of genes involved in the pathogenicity of *Fusarium oxysporum* [23]. However, attributes such as growth; asexual development; and processes related to infection, pathogenicity, and tolerance to abiotic stress have been evaluated and, on occasion, no significant differences were observed between deletion transformants and isolates of type wild [14]. For instance, the MoIRR-targeted knockout transformants did not show significant differences in mycelial growth, conidial production, conidial germination, or pathogenicity compared with *M. oryzae* parental isolates. MoIRR is a Zn2Cys6 transcription factor with conserved domains similar to GAL4 and Fungal_TF_MHR present in some of the well-studied Zn2Cys6 transcription factors. The genetic transformation verified that the variation in the MoIRR gene was associated with resistance to the IPT fungicide [24]. In *P. digitatum*, different Zn_2_Cys_6_ transcription factors were identified. PdPacC mediates pH modulation and is also involved in the pathogenicity of *P. digitatum* [25], a calcineurin-sensitive transcription factor PdCrz1 that is related to the pathogenicity of *P. digitatum* through the positive regulation of the cell wall synthase genes that maintain the cell wall integrity of *P. digitatum* [26], and PdSte12 controls invasive growth and asexual reproduction, thus affecting virulence [8,12].

In this work, we have identified PdMut3, a Zn_2_Cys_6_ transcription with conserved GAL4-like and Fungal_TF_MHR domains, and, through evaluation of deletant mutants, we have tried to clarify the role it exerts during the fruit–pathogen interaction and what its contribution could be to the virulence or sensitivity to certain compounds.

## 2. Materials and Methods

### 2.1. Fungal Strains’ Growth and Transformation

Two *P. digitatum* isolates, Pd1 (CECT20795) and Pd149 (CECT2954), were used in this work, as already described [7].

Depending on the further use, all fungi were grown in either potato dextrose broth (PDB; Liofilchem Laboratories, TE, Italy) or potato dextrose agar (PDA; Liofilchem Laboratories, TE, Italy). Fungal cultures were incubated at 25 °C with continuous light for 1 to 3 days (liquid cultures) or 1 week in the dark (solid media). Spores were obtained from 1-week-old PDA plates and conidia were counted with a hemacytometer and prepared to a desired final concentration.

*P. digitatum* transformation was achieved with *Agrobacterium tumefaciens* C_58_C_1_. *A. tumefaciens* containing plasmid construct was grown in LB plates or LB liquid medium with 50 µg/mL Rifampycin and 100 µg/mL Kanamycin at 28 °C, as previously reported [8].

### 2.2. Cloning and Targeted Gene Disruption

Genomic DNA was isolated from *P. digitatum* mycelium, as reported by Marcet-Houben et al. [27]. All PCR amplicons described in this work were purified using Ultra Clean TM PCR Clean-up (MoBio, Solan Beach, CA, USA). Gene identity and gene cloning verification was done by DNA sequencing [28]. DNA sequences were compared with those from the EMBL database with the Washington University Basic Local Alignment Search Tool (WU-BLAST) algorithm [29].

A binary plasmid pRFHU2 [30] was used for gene disruption following a similar procedure as described previously [9].

### 2.3. Protein Characterization

The protein domains were analyzed using SMART (http://smart.embl-heidelberg.de, 27 of July 2021) and fungal domains were predicted using InterPro Scan search (http://www.ebi.ac.uk/interpro/search/sequence/, 27 July 2021), which showed domains and the prediction of cellular localization.

Sequence protein alignments of different *P. digitatum* transcription factors, including the new gene reported, were performed using the Clustal Omega (https://www.ebi.ac.uk/Tools/msa/clustalo/, 27 September 2021), and the phylogenetic tree was constructed using the Mega 7.0 program with the maximum likelihood method.

### 2.4. Molecular Characterization of ΔPdMut3

Gene removal was confirmed with three independent PCR reactions: (1) amplification from the 5’ flank of each gene to *hph* gene, (2) amplification from the 3’ flank of each gene to *hph* gene, and (3) amplification of the coding region of the gene.

The evaluation of gene copy number was done using real-time quantitative PCR (qRT-PCR), with beta-tubulin as the reference gene [9].

### 2.5. Inhibition of Mycelial Growth In Vitro

Four fungicides commonly used during citrus postharvest handling were used for sensitivity evaluation: imazalil (IMZ) (Textar I; Tecnidex, Valencia, Spain), prochloraz (PCL) (Ascurit; Tecnidex), and philabuster (a mixture of imazalil and pyrimethanil) (PHI) (Decco Ibérica), at increasing concentrations of 0, 1, 2, 4, and 10 µg/mL, and thiabendazol (TBZ) (Textar 60 T; Tecnidex) at 0, 10, 20, 40, and 100 µg/mL. Analysis was done in triplicates in two independent experiments. The assessment of mycelial growth at different chemicals’ concentrations was performed in 96-well microtiter plates and the respective untransformed wild-type strains were assayed at the same time. Sensitivity to chemical compounds was computed as relative mycelial growth, expressed as a percentage, calculated by comparing growth in absence and presence of the chemical following the protocol previously defined [31].

Sensitivity tests were assayed transferring 5 µL of 10^4^ conidia/mL to PDA containing a test compound and incubation at 25 °C in the dark. Tested compounds included methanol (2.5%), FeCl_3_ (0.2 mM), EDTA (0.05 mM), SDS (0.02%), Tween 20 (T-20, 0.5%), 1 mM H_2_O_2_, calcofluor white (CFW, 200 µM), and Congo red (CR, 50 µM). Fungal radial growth was measured at 4–7 days. Each treatment was performed in three replicates and experiments were repeated three times. Relative growth was calculated by comparing the difference in the growth of the Pd1 and the mutants expressed in percentage.

### 2.6. Pathogenicity Evaluation of ΔPdMut3 Mutants

‘Navel’ mature oranges without chemical treatments were used for in vivo assays and fruit inoculation was performed as reported previously [10]. Pathogenesis experiments were performed using freshly harvested oranges (*Citrus sinensis*) that were injured at four places around the equatorial axis and infected with 10 µL of a conidia suspension adjusted to 10^5^ conidia/mL. They were kept at 20 °C and 90% RH. Three replicates of five fruits each were performed and the infection experiments were performed twice. As control, mocked-inoculated fruits were used. Infection progression was computed using two parameters: percentage of infected fruits (disease intensity) and diameter of macerated tissue (disease severity).

### 2.7. RNA Extraction and Relative Expression by RT-qPCR

The trizol method (Ambion Inc., Austin, TX, USA) was used for RNA extraction from *P. digitatum* frozen mycelium. The extraction of total RNA from infected samples was processed as reported previously [6].

PrimeScript™ RT reagent Kit (Takara Bio Inc., San Jose, CA, USA) was used for synthesis of the first strand of cDNA in a 20 µL reaction, following the instructions of the manufacturer. Quantitative PCR was performed as stated before [8].

Experimental values obtained were an average of two repetitions of three biological replicates. Oligos qMut3F and qMut3R were used for *PdMut3* gene, and genes coding for fungal µ-tubulin (qTubF-qTubR), ribosomal protein 28S (q28SF-q28SR), and histone H3 (qH3F-qH3R) were independently used as reference genes (Table S1). LightCycler 480 Software, version 1.5 (Roche Diagnostics) was used for cycle quantification. Primer melting temperature allowed the selection of each primer set for specific amplification. Relative gene expression was carried out as previously described [7]. PrimeScript™ RT reagent Kit (Takara Bio Inc.) was used for synthesis of the first strand of cDNA in a 20 µL reaction, following the indications of the manufacturer. Quantitative PCR was performed as reported before [8]. The software LightCycler 480 SW 1.5 (Roche Diagnostics) was used for cycle point quantification. Primer melting temperature allowed the selection of each primer set for specific amplification. The relative gene expression (‘RGE’) was carried out as stated before [7].

### 2.8. Microscopic Visualization

Each fungal sample was stained with 50 µg/mL Calcofluor White (CFW) for 5 min in the dark. The fluorescence was examined and photographed by a Nikon E90i fluorescence microscope (Nikon Corporation, Tokyo, Japan) with DAPI filter sets. Fluorescence images were obtained by the NIS-Elements BR v2.3 software (Nikon) and processed using FIJI software [32].

### 2.9. Statistical Analysis

Significant differences were evaluated using analysis of variance (ANOVA) with SAS software (SAS Institute Inc., Cary, NC, USA). Statistical significance was defined as *p* < 0.05; when the analysis was statistically significant, Tukey’s test for separation of means was performed.

## 3. Results

### 3.1. Identification of P. digitatum PdMut3

Specific oligos (Table S1) were used based on partial sequence of a putative transcription factor gene available in our group. Primers Mut-1 and Mut2 were used to screen Pd1 genomic library. The genomic library search was carried out as reported before [7].

After complete sequence analysis, *PdMut3* (PDIP_75320) was identified. *PdMut3* presented an open reading frame of 3279 bp that was interrupted by four introns of 107, 54, 58, and 51 bp, placed at positions 1.. 197, 305.. 1072, 1127.. 1738, 1797.. 2181, and 2233.. > 3279 of the coding region. The deduced amino acid sequence encoded a protein of 1002 amino acids. The protein domains analyzed using SMART showed GAL4-like and Fungal_TF_MHR domains also predicted by InterPro Scan that illustrated domains and the prediction of cellular localization.

**Figure 1:**
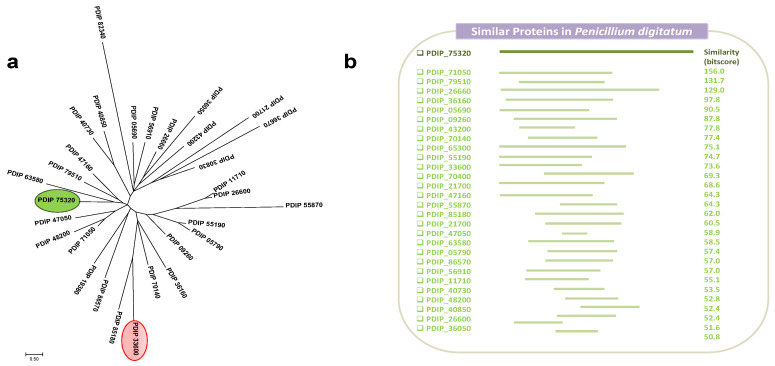
(**a**) Unrooted maximum likelihood phylogenetic tree of selected *P. digitatum* TF proteins. PdMut3, the new TF described in this study, is highlighted in green, and in red, a TF found in an SSH cDNA library related to *P. digitatum* virulence. Each branch topology was found 100% of the time during bootstrap analysis. The tree is drawn to scale, with branch lengths measured in the number of substitutions per site. NCBI accession number is indicated for each TF protein. (**b**) Set of proteins within *P. digitatum* Pd1 that encode the same type of transcription factor corresponding to type III. The regions of greatest similarity are indicated.

Sequence protein alignments of different *P. digitatum* transcription factors, including the new gene reported, were performed using the Clustal Omega (https://www.ebi.ac.uk/Tools/msa/clustalo/, 27 September 2021), and the phylogenetic tree was constructed using the Mega 7.0 program with the maximum likelihood method (Figure 1a). Phylogenetic study shows the existence of numerous TFs that are distributed randomly in the tree and where there is no single cluster with attributed functions in a specific way. In fact, PdMut3, highlighted in green, does not cluster with another *P. digitatum* TF (PDIP_33600), highlighted in red, which was identified in a cDNA library constructed based on increased virulence. PDIP_33600 gene showed a high rate of transcription (fourfold compared with in vitro growth) during citrus infection, especially at early stages [33].

Protein searching analysis showed that at least 26 TFs are present in *P. digitatum* that share the same typical structure with GAL4-like and Fungal_TF_MHR domains. The evaluation of the similarity between all these proteins reflected large differences between all the proteins when compared with PdMut3 (Figure 1b). Although these proteins are putative TFs and possess the GAL4-like and Fungal_TF_MHR domains, the function exerted by each of them has not been determined, nor in the signaling pathways in which they are involved.

### 3.2. Construction and Characterization of Deletion Mutants

**Figure 2:**
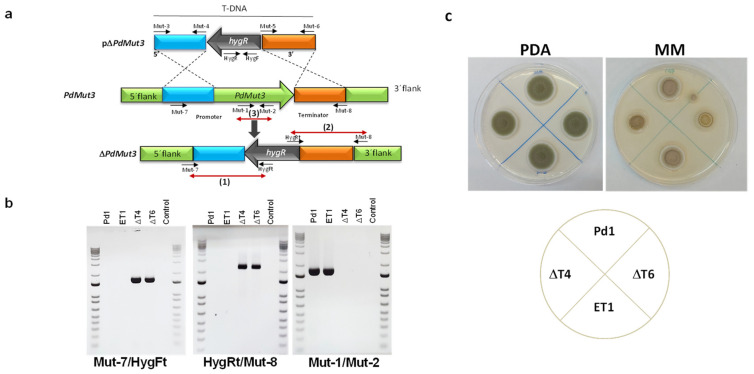
(**a**) Diagram of wild-type locus and the *PdMut3* replacement with the HygR selectable marker from pΔ*PdMut3* by homologous recombination to generate the ΔMut3 mutants. Red arrows define PCR fragments used for mutants’ confirmation (**b**) Polymerase chain reaction (PCR) evaluation of the wild-type Pd1 strain, two Δ*Mut3* null mutants (ΔT4, ΔT6), and the respective ectopic mutant (ET1) with analytic primers. (**c**) Mycelial growth of parental strain Pd1 and mutants in potato dextrose agar (PDA) and minimal medium (MM) during 4 days at 25 °C.

*A. tumefaciens*-harboring plasmid pΔMut3 (Figure 2a) was used for *P. digitatum* Pd1 transformation. The pΔ*PdMut3* contained a 1.6 kb fragment from the upstream gene region amplified using the Mut-3/Mut-4 primers and a 1.8 kb fragment from the downstream gene region amplified using the Mut-5/Mut-6 primers (Table S1) adjacent to the hygromycin resistant cassette in the T-DNA region of the plasmid (Figure 2a).

Deletion of the targeted gene was analysed from both flanks: (1) 5′ flank with primers Mut-7/HygFt and (2) 3′ flank with primers HygRt/Mut-8 (Table S1), confirming the presence of either 5′ flank plus promoter or 3′ flank plus terminator next to the hygromycin gene (Figure 2b). Bands were only present in deletion mutants. (3) The removal of *PdMut3* gene was verified by PCR with primers Mut-1 and Mut-2. Bands were only present in the Pd1 wild-type and in ectopic transformants and were absent in deletant transformants and negative controls (Figure 2b).

To select those null mutants without additional T-DNA integrations, the copy number of integrated DNA was determined by real-time quantitative PCR (RT-qPCR). Two knockout mutants (ΔT4 and ΔT6) harboring only a single T-DNA integration and one ectopic mutant (ET1) were selected for further analysis.

Phenotypic traits of the different strains WT, deletant transformants (ΔT4 and ΔT6), and ectopic transformant (ET1) did not show differences when grown on PDA regarding growth or the rate of sporulation, but differences were observed in minimal media where deletion mutants had 35% reduced mycelial growth (Figure 2c).

### 3.3. Involvement of PdMut3 in Fungal Infection

Infection of different fungal strains was conducted in mature orange fruits to define if *PdMut3* gene displayed a role in pathogenicity/virulence.

**Figure 3:**
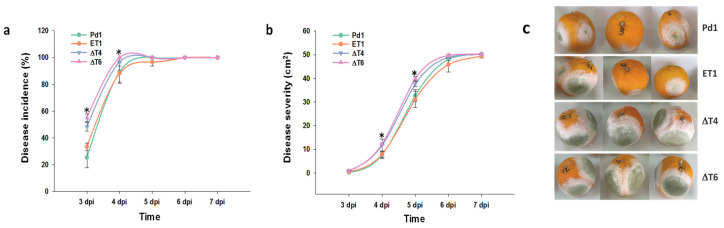
Analysis of fungal infection. (**a**) Disease incidence (%) and (**b**) disease severity (cm^2^). Virulence assessment of Pd1, ectopic transformant ET1, and disruptant transformants (ΔT4, ΔT6). All are means of two infection experiments. Error bars represent standard deviation. * Significant differences between treatments using Tukey’s test (*p* < 0.05) at each dpi. (**c**) Representative images of infected oranges at 5 dpi.

The analysis of the infectivity of the mutant strains against the wild-type Pd1 showed that the ectopic mutant behaved in a similar way to Pd1 strain, while surprisingly, an increase in the infectivity was observed in all deletant mutants. Although the rise was significant at early stages 3–4 dpi, no differences were observed from this point. Disease severity was also affected, but differences were observed at later time points (Figure 3a,b).

### 3.4. PdMut3 Has No Contribution to Fungicide Resistance

**Figure 4:**
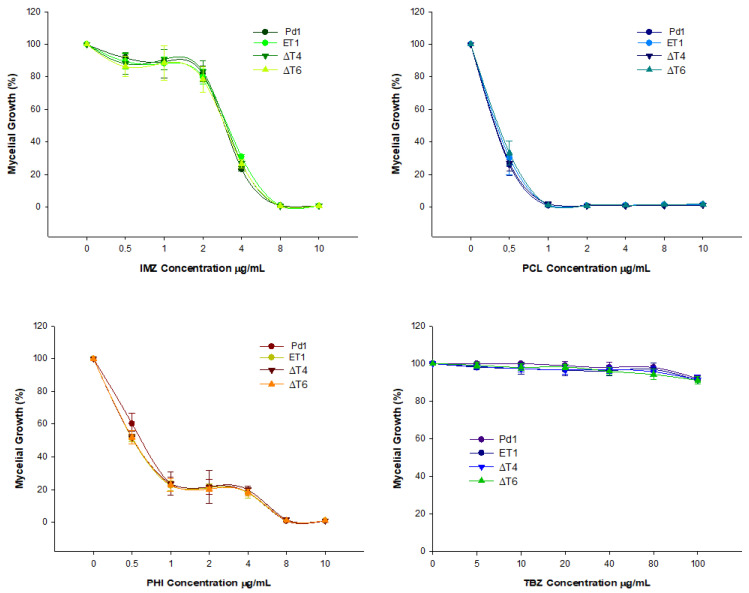
Estimation of fungicide sensitivity in disruptant mutants compared with wild type Pd1. IMZ = imazalil, PCL = prochloraz, PHI = Imazalil + pyrimethanil, and TBZ = thiabendazol. The fungicides’ concentration is expressed in Δg/mL. Percentage of relative growth was calculated with respect to each strain grown without fungicide. Error bars represent standard deviation among three replicas. * Significant differences between treatments using Tukey’s test (*p* < 0.05).

Determination of the effect of several fungicides was carried out in the different deleting transformants. Elimination of *PdMut3* gene did not affect the sensitivity of any particular chemical regardless of the type of fungicide. The results showed that all mutant strains exhibited an identical profile, which was similar to Pd1 (Figure 4).

### 3.5. PdMut3 Is Involved in the Maintenance of Cell-Wall Integrity

**Figure 5:**
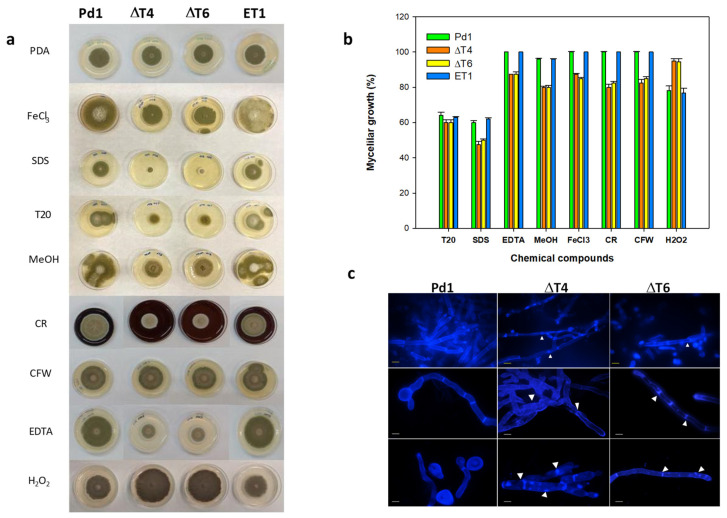
(**a**) Fungal growth of Pd1, ET1, and ΔPdMut3 mutant (ΔT4, ΔT6) on PDA amended with different chemical compounds during 5 days: FeCl_3_, SDS, Tween 20 (T20), methanol (MeOH), Congo red (CR), calcofluor white (CFW), EDTA, and H_2_O_2_. (**b**) Percentage of growth reduction of ΔPdMut3 in relation to that grown on PDA amended with different concentrations of chemical compounds after 7 days. (**c**) The effects on the cell wall of spores and mycelia of *P. digitatum* strains observed under a fluorescence microscope after staining with calcofluor white (CFW). White arrows point out strangulation in the hyphae and variation in the septum. Yellow bars correspond to 20 µm and white bars to 40 µm.

Evaluation of mycelial growth on different chemical compounds showed that Tween 20 (a polysorbitan containing lauric acid) affected all fungal strains in a similar manner by causing a 40% growth reduction. Conversely, Δ*PdMut3* transformants displayed reduced radial growth by nearly 15% on PDA containing EDTA (0.05 mM), and 20% on methanol (2.5%), FeCl_3_ (0.2 mM), calcofluor white (CFW), and Congo red (CR). The addition of SDS (0.02%) affected Δ*PdMut3* mutants drastically by inhibiting their growth, although Pd1 and ET1 also presented growth reduction (Figure 5a,b). Treatment with H_2_O_2_ confirmed that deletant mutants were able to grow better than the wild-type, being more resistant to ROS.

Changes in mycelial morphology of deletion mutants were analyzed by fluorescence microscopy under CFW staining that binds chitin. The mycelium of both deletant mutants ΔT4 and ΔT6 exhibited strong microscopic alterations (Figure 5c). The areas of the septum were thinner and more diffuse and, in some cases, showed strangulation in the hyphae. Mutants exhibited intense CFW staining and occasionally broke down and released the intracellular content (Figure 5c).

### 3.6. Transcriptional Profiling of PdMut3

**Figure 6:**
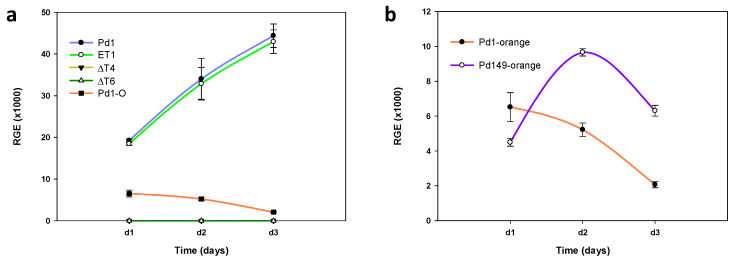
Analysis of *PdMut3* relative gene expression (RGE). (**a**) Time course evaluation of gene expression of Pd1, ectopic mutant ET1, and deletant mutants (ΔT4 and ΔT6) grown in PDB liquid culture at 25 °C and time course comparison of gene expression of Pd1 during orange infection. (**b**) Time course evaluation of gene expression of Pd1-virulent *P. digitatum* strain and Pd149-low virulent strain during orange infection. In all cases, d1, d2, and d3 correspond to 1 dpi, 2 dpi, and 3 dpi, respectively. The expression levels are relative to three reference genes: ribosomal 28S RNA, β-tubulin, and histone H3. Error bars indicate standard deviations of three biological replicates.

Examination of *P. digitatum* transcription factor gene expression was conducted using RT-qPCR. As shown in Figure 6a, gene transcription in axenic growth increased over time. No discrepancies were observed between parental strain Pd1 and the ectopic strain, while both deletant mutants assayed exhibited no expression at all.

Comparison of *PdMut3* gene expression in Pd1 strain in vitro and in vivo revealed that the expression dropped drastically during infection. Furthermore, the transcription tendency was just the opposite to that observed in vitro and the expression during the infection progressively decreased over time (Figure 6a).

Given the results observed during infection, a comparison was made of two isolates of *P. digitatum* that vary in their degree of virulence. The results obtained during infection (Pd149) showed transcription around two times higher at 48 h after infection compared with Pd1, and the transcription decreased with time, but maintained the difference with respect to the virulent strain (Figure 6b).

## 4. Discussion

*P. digitatum* is one of the most relevant pathogens responsible for postharvest losses in citrus [1]. The study of fruit–pathogen interactions has shown greater interest and advances have been made in the knowledge of the pathogenicity of fungi. In the citrus–*P. digitatum* interaction, the availability of the entire genome has greatly facilitated a better understanding of the pathogenicity of *P. digitatum* at molecular levels [33].

In this work, we addressed the identification of *PdMut3* that encodes a Zn_2_Cys_6_ transcription factor with conserved GAL4-like and Fungal_TF_MHR domains. Remarkably, it should be noted that the most relevant pathogenicity elements described to date, which are also responsible for the virulence of *P. digitatum* in citrus, include mainly transcription factors, enzymes related to the cell wall, protein kinases, and fungal transporters [5]. The phylogenetic analyses carried out made it possible to determine the presence of a large number of transcription factors with similar topology that could not be grouped around PdMut3. Furthermore, another transcription factor identified in a virulence gene-enriched cDNA library that showed high gene transcription during citrus infection [33] is phylogenetically distant from PdMut3. Therefore, it is difficult to establish a clear function for the different transcription factors.

The functional activity of PdMut3 has been achieved by gene elimination. The deletion mutants were confirmed molecularly and, from the point of view of their axenic growth, differences in growth on solid media were observed. The mutants presented decreased growth on minimal media, but not in PDA, suggesting that metabolism is likely affected. In addition, the growth of the mutants in PDA medium supplemented with different compounds showed that the elimination of *PdMut3* affects the cell wall integrity. A decrease in mycelial growth in the presence of CFW and CR was observed and growth was also affected in the presence of methanol, FeCl_3_, and chelators such as EDTA, indicating that metabolism is altered.

The increase in CFW sensitivity is indicative of alterations in the cell wall and chitin content. Damage to the cell wall structure was confirmed by microscopic studies performed with CFW staining, where the mutants showed cell disorganization, strangulation in hyphae, and chitin deposits. These effects have already been described in other cases where the cell wall was clearly damaged, for instance, in *PdCrz1* mutants [26]. This result was also similar to the one observed with the addition of antifungal proteins on cell wall chitin [34]. The cell membrane plays a relevant role in maintaining cell viability because it represents a barrier by separating the cell from its environment, and is also a means of exchange of substances and energy between the cell and the surrounding environment. Generally, its integrity is highly related to many metabolic processes [35].

In this study, we evaluated the possible implication of *PdMut3* in the sensitivity to certain fungicides owing to the detected alteration of the cell wall and because many TFs are involved in stress responses and pleiotropic drug resistance [20]. Previous studies performed with MoIRR, a Zn_2_Cys_6_ transcription factor of *M. oryzae*, which has the conserved GAL4-like and Fungal_TF_MHR domains, revealed that the knockout of MoIRR gene did not exhibit relevant differences attributable to variations in pathogenicity/virulence pathogenicity compared with *M. oryzae* parental isolates, but MoIRR gene was associated with resistance to IPT fungicide [24]. However, the tests carried out in *PdMut3* mutants showed that this gene does not have a relevant role in the resistance to fungicides and knock-out mutants showed exactly the same resistance pattern for the four chemical compounds tested. This effect is in agreement with a study carried out with the C6 transcription factor *PdSte12*, which showed that it does not intervene in fungicide activity through triggering of a fungal signaling pathway [8].

In the search for the role performed by *PdMut3*, infective capacity studies were carried out in oranges with knockout mutants. The results were very surprising and, instead of showing a decrease in virulence, as might be expected for a relevant role in pathogenesis/virulence, just the opposite was detected. The Δ*PdMut3* mutants showed a higher infective capacity during the initial stages, as demonstrated by both the incidence and the severity of the disease, particularly in the early stages. It is well known that the progression of *P. digitatum* during infection includes spore germination, germ tube, growth, differentiation of conidiophores stems, and formation of phialids and new conidia [36]. Therefore, initial states of infection are crucial. Previous studies have shown that several genes control the different steps of growth and development of fungi, and the elimination of any of these genes leads to growth defects and loss of pathogenicity of pathogens [2]. Nevertheless, in the RNA-seq studies performed in order to identify genes involved in virulence in *P. digitatum*, *PdbrlA* (PDIP_05330), a C_2_H_2_ zinc-finger transcription factor, was down-regulated, while the genes *PdVEA1* (PDIP_21430) and *PdVelB* (PDIP_64730) were up-regulated, being related not only to fungal development, but also to oxidative stress sensitivity [37].

The elucidation of the possible function of the *PdMut3* gene led us to study its expression pattern. In in vitro studies, the expression rate showed an increasing level over time. According to what was seen in the infection assays, it was observed that the wild-type strain Pd1 had a much lower level of expression during infection, which confirms that it does not play a role in the infectious capacity in a direct way. This is contrary to what was previously described for other TFs such as *PdPacC* [25], *PdCrz1* [26], *PdsreA, PdsreB* [38,39], or *PdSte12* [8,12], which all contribute in some way to the virulence of *P. digitatum*.

Previous works showed that Mut3p protein has been associated with peroxisomes and methanol metabolism [40]. Multiple metabolic processes occur in peroxisomes, thus they play a crucial role in the development and pathogenesis of fungi and virulence [41,42,43,44,45]. For instance, peroxisomal metabolic function is required for appressorium-mediated plant infection by *Colletotrichum lagenarium* [42], peroxisomal fatty acid-oxidation occurred during plant infection in *M. grisea* [44], and peroxisome functions are of vital importance to pathogenesis of the tangerine pathotype of *Alternaria alternata* [45]. Taking all this together, it could be possible that *PdMut3* plays an indirect role in fungal infectivity through control of metabolism, as deletion of this gene leads to increased infectivity. We suggest that *PdMut3* could play a role in the control of peroxisome development, probably by means of a negative control that promotes its degradation.

Peroxisomes are small conserved organelles that carry out a variety of critical functions. In fact, peroxisomes participate in the uptake of reactive oxygen species (ROS), through catalases and peroxidases, which are abundant in peroxisomes [46]. In *Hansenula polymorpha*, peroxisome degradation resulted in increased ROS formation [47]. In the early stages of infection, the oxidative stress sensitivity should occur in *P. digitatum*, when the citrus fruit has a series of oxidative bursts in response to infection [48]. Our hypothesis would be that, if *PdMut3* regulates the degradation of peroxisomes, the knockout mutants would allow the increase of catalases, helping the degradation of the H_2_O_2_ produced as a defense response of the host [49] and improving the infectivity of *P. digitatum*, as has been observed in deletant mutants. This would be in agreement with the results obtained in the presence of H_2_O_2_, where the deletant mutants were able to grow better than the wild-type Pd1. This effect was also supported by the higher transcription rate shown in the less-virulent strain (Pd149) during infection.

Moreover, in fungi, peroxisomes have been shown to be required for sexual and asexual reproduction [50]. Different TFs appear to be involved in the regulation of peroxisomal proteins [40]. A correlation between *PdMut3* and *PdSte12,* clearly involved in initial stages of *P. digitatum*–citrus infection and impaired in asexual reproduction during orange fruit infection [8], was established. In previous studies, we showed that *PdSte12*-disruptant mutants displayed increased *PdMut3* gene expression of 3–4-fold [12], so *PdSte12* could negatively regulate *PdMut3.*

The involvement of peroxisomes in cell wall integrity has also been reported in other fungal pathogens such as *Fusarium graminearum, M. oryzae,* and *A. alternata* [45,51,52,53]. As described above, in this study, deletant mutants showed a clear effect on cell wall integrity, which indicates that peroxisomes metabolism could be altered, affecting infection capacity.

Nevertheless, it would be necessary to go deeper into the process that regulates the expression of the numerous genes involved in the proliferation and degradation of peroxisomes to determine the relative contribution of *PdMut3* to each of them. More research will be needed to understand all the processes in which peroxisomes are involved that likely will shed some light on how *P. digitatum* responds to stress and nutrient availability during penetration and colonization of citrus fruits.

## 5. Conclusions

To improve the postharvest disease control technology of citrus fruits, it is important to explore fungal infection mechanism. The infection mechanisms between *P. digitatum* and citrus depend on several factors that mediate and affect this interaction.

This study provides the identification and characterization of *PdMut3*, a new Zn2Cys6 transcription factor with conserved GAL4-like and Fungal_TF_MHR domains of *P. digitatum*. Functional analysis carried out by gene elimination confirms that *PdMut3* in not involved in fungicide sensitivity, but appears to be indirectly implicated in fungal virulence. Mutants demonstrate higher infectivity in citrus fruit, although this was not correlated with a higher gene transcription rate. Our hypothesis is that *PdMut3* could be related to metabolism through peroxisomes development, regulating their degradation, and could also be negatively controlled by *PdSte12* transcription factor involved in the Fus3 MAPK metabolic pathway.

## Supplementary Materials

The following are available online at https://www.mdpi.com/article/10.3390/jof7100828/s1, Table S1: Oligo sequence used in this study.
