# Supplementary material for: Unveiling the Role Displayed by Penicillium digitatum PdMut3 Transcription Factor in Pathogen–Fruit Interaction
Source: J Fungi (Basel). 2021 Oct 3;7(10):828. doi: 10.3390/jof7100828 (PMC8540835; doi:10.3390/jof7100828)
Supplement: Supplementary file 1 [file jof-07-00828-s001.zip › jof-1409199-supplementary.pdf]

Table S1. Oligo sequence used in this study.

Name	Sequence (5′-3′)
Mut-1	AGTCCTATGCAGTTAATCTCGC
Mut-2	ACAGGCATACGACCGCTTATCCA
Mut-3	GGTCTTAAUTAGAGCGGAATAGTCAAGAGG
Mut-4	GGCATTAAUATTTGGGATCTGTGGTGGAG
Mut-5	GGACTTAAUAGAGCCATACTCCATCAGACG
Mut-6	GGGTTTAAUTAGCCCTGCCTGGTTAGAC
Mut-7	AGTCGCGTTGGCGTTGTTATAC
Mut-8	GCCTTCATCATTCTCACTGC
HygRt	ATCGAAGCTGAAAGCACGAG
HygFt	GGCAATTTCGATGATGCAGC
HygR	AGCTGCGCCGATGGTTTCTACAA
HygF	GCGCGTCTGCTGCTCCATACAA
hTubF	AGCGGTGACAAGTACGTTCC
hTubR	ACCCTTAGCCCAGTTGTTAC
qTubF	AGCGGTGACAAGTACGTTCC
qTubR	ACCCTTAGCCCAGTTGTTAC
q28SF	TTATAGCCGAGGGTGCAATG
q28SR	TTTCAAGACGGGTCGCTTAC
qH3F	AGGCTCCCCGTAAGCAGCTCGC
qH3R	CGACATGAGGCGGAACTTACCGG
qMut3F	AGTTGCAGCTGCGTACGATG
qMut3R	ACCAAATTGCCGAGACCACG
